# Perceived Social Support Among People With Physical Disability

**DOI:** 10.5812/ircmj.12500

**Published:** 2013-08-05

**Authors:** Ameneh Setareh Forouzan, Abolfazl Mahmoodi, Zahra Jorjoran Shushtari, Yahya Salimi, Homeira Sajjadi, Zohreh Mahmoodi

**Affiliations:** 1 Social Determinant of Health Research Center, University of Social Welfare and Rehabilitation Sciences, Tehran, IR Iran; 2 Faculty of Medicine, Shahid Beheshti University of Medical Sciences, Tehran, IR Iran; 3 University of Social Welfare and Rehabilitation Sciences, Tehran, IR Iran; 4 Department of Epidemiology and Biostatistics, Public Health School, Kermanshah University of Medical Science, Kermanshah, IR Iran; 5 Department of Epidemiology and Biostatistics, Public Health School, Tehran University of Medical Science, Tehran, IR Iran

**Keywords:** Perceived Social Support, Physically Disabled, Welfare Organization, Iran

## Abstract

**Background:**

Disability is more based on social, rather than medical aspects. Lack of attention and social support may impact on participation of people with physical disability in various aspects and their return to normal life in the society.

**Objectives:**

This study was conducted to determine perceived social support and related factors among physically disabled in the city of Tehran.

**Patients and Methods:**

This cross-sectional study by using simple random sampling was conducted on 136 people with physically disabled who were covered by Welfare Organization of Tehran. The Norbeck social support questionnaire was used .Multiple linear regression analysis with the backward method was used to identify the adjusted association between perceived social support as dependent variable and demographic variables as independent variables.

**Results:**

The present sample comprised of 68 (50%) male and 68 (50%) female with the mean age of 33 (SD = 8.9) years. Based on the results, mean of functional support was 135. 57 (SD = 98.77) and mean of structural support was 77.37 (SD = 52.37). Regression analysis model, demonstrates that variables of age and marital status remained in the model as significant predictors of functional support (P = 0.003, P = 0.004, respectively) and structural support (P = 0.002, P = 0.006, respectively).

**Conclusions:**

Based on the results, participants in the study didn’t have favorable status with respect to perceived social support (in all dimensions) from their social network members. While, social support as one of the social determinants of health, plays an important role in improving psychological conditions in people’s lives; therefore, being aware of social support and designing effective interventions to improve it for the disabled is very important.

## 1. Background

It has been reported that more than 10% of population in the world suffer from physical, mental, and social disabilities. Of these people, 80% live in the developing countries, and one third are children (1). Therefore, it is expected that nearly 7 million people live with various disabilities in Iran. Disability is complex, dynamic and multidimensional phenomenon that is more based on social rather than medical aspects (1). Over recent decades, several researchers from the social and health sciences have identified the role of social and physical factors in disability (2, 3). Although disability and its consequences such as inability, depression and isolation are considered as stressful and aggravating conditions, they are not the unavoidable consequences for disabled people (1). The problems which disabled people are faced with are not necessarily due to their mental and physical disability but traditional attitudes of the society (4) and lack of attention to their physical and psychosocial characteristics may affect mental health and their participation in various aspects of social and personal life (5). Their problems depend on the strength and efficacy of coping methods, especially social support (6). Lack of attention and support of disabled people may effect on quality of life and increase the problems of this vulnerable group (7). Social support emphasizes relationship with whom that provides support and availability of support resources when are needed (8). Social support creates mutual obligations, in which, an individual feels loved, cared for and valued (8, 9). According to the social support theories, relationships are not necessarily sources of social support unless the people perceive them as available and suitable sources of support for their needs (10). The psychosocial benefit of social support among disabled people may be due to its effects on their mental evaluation of pressure factors, choosing effective coping methods, improving self-esteem, personal skills, better social life, and empowering them for help in the process of social development (4, 11). Then social support, as a social determinant of health, plays an important role in enhancing psychosocial conditions in people’s lives (5, 12, 13). Despite the increasing body of literature on the concept of social support and its association with health and psychological well-being in different populations (14-16), there is little information on the disabled people in Iran.

## 2. Objectives

This study was conducted to determine the status of perceived social support and related factors among the physically disabled who covered by Welfare Organization of Tehran city.

## 3. Patients and Methods

This cross-sectional study was conducted among people with physically disabled who covered by the Welfare Organization of Tehran, Iran (Shemiranat, Tehran province, south) in 2011. Of these people, 136 completed Norbeck social support questionnaire. Sampling method was simple random sampling that was performed from a list of physically disabled people. Given the importance of gender variable in social support (based on the literature review) the subjects based on the gender among the list of physically disabled people were classified and each class (male, female) was numbered. After that participants randomly selected with using table of random numbers. Based on inclusion criteria in the present study, respondents had to be over 15 years of age, being literate, having physical disability since at least six months ago. Because the duration of time (of the point of diagnosis as disability) may need to effect on the social support network, six months has been chosen. People with Intellectual Disabilities, blindness and illiterate people because of self-administered questionnaires were excluded. Prior to the study, participants signed an informed consent form after they were informed of the objectives of the study and were assured that their information would remain confidential and they could withdraw from the study whenever they liked.

The self-administrated Norbeck social support questionnaire (NSSQ) was used to measuring perceived social support. This Questionnaire is including of some subscales as emotional support, instrumental support, functional support (sum of Emotional and instrumental support; range = 0 - 384), Structural support (sum of network size, duration and frequency of contact; range = 0 – 240) and Loss support (0-120). But the sub scale of “total Loss” was eliminated because participants had varied responses with too missing, which could not be reported. The reliability and validity of the NSSQ were confirmed in other studies (17-19). Reliability and validity of the Farsi version of the NSSQ have been evaluated and approved (20). In the present study, Cronbach's Alpha scores for sub scales have ranged from 0.86 to 0.95.

Additionally, a socio-demographic checklist was used. It was including of questions on gender, age, marital status (single, married, widowed, divorced), level of education (high school dropout, high school diploma and higher), employment status, network size, severity of disability, insurance, and socioeconomic status. The questionnaire and checklist were completed in self-administered form. To determine adjusted association between perceived social support (Structural and functional support) as dependent variables and socio-demographic variables as independent variables, multiple linear regression model (considering the establishment of the required assumptions) with backward method was used. The variables of sex, age, marital status, educational level, employment, network size, severity of disability, insurance, and socioeconomic status were used as independent variables. The level of education was entered into the model as an indicator variable. The baseline for this variable was considered high school dropout (21). After logarithmic transformation of dependent variables (functional and structural support), Kolmogorov-Smirnov Test was used to check normality assumption (respectively, P = 0.987 and P = 0.35). Data were analyzed by SPSS 11 software. The significance level was at 0.05.

## 4. Results

Of the 136 participants, 68 (50%) were male and 68 (50%) were female, with the mean age of 33 (SD = 8.9) years. The majority of the participants were single 64.7% (Table 1). 

**Table 1.** Frequency Distribution of Demographic Details of Study Participants

	Mean, (S.D) or No, (%)
**Network size, Mean, (SD)**	8.08 (5.28)
**Gender, No., (%)**	
Male	68 (50%)
Female	68 (50%)
**Marital status, No., (%)**	
Married	16 (11.8%)
Single	88 (64.7%)
Widowed	13 (9.6%)
Divorced	16 (13.8%)
**Employment, No., (%)**	
Unemployed	91 (66.9%)
Employed	45 (32.3%)
**Education level, No., (%)**	
High school dropout	69 (50.7%)
High school diploma	44 (32.8%)
Graduate	23 (16.9%)
Mild	17 (12.5%)
Moderate	47 (34.6%)
**Severity of disability, No., (%)**	
Severe	48 (35.3%)
Very severe	24 (17.6%)
**Insurance, No., (%)**	
Covered	107 (78.7%)
Not covered	29 (21.3%)

As shown in Table 2 social support was considered in two different types of functional and structural support. The mean of functional support was 135.57 (SD = 98. 77) and the mean of structural support of 77.37 (SD = 52.37).

**Table 2.** Social Support Status Sub-Scales among Participants

Variables	Mean (SD)	Min - max	Attainable range
**Functional support**	135.57 (98.77)	17 - 565	0 – 576
**Emotional support**	90.24 (64.54)	12 - 377	0 – 384
**Instrumental support**	45.33 (33.1)	5 - 189	0 – 192
**Structural support**	77.37 (52.37)	7 - 392	0 – 240

In the bivariate analysis, there were significant associations between network size (P = 0.032) and gender (P = 0.011) with functional support. Also, Structural support had significant association with education level (P < 0.001) and network size (P = 0.013). Table 3 and Table 4 present variables that remained in the final regression model with using the backward method. It can be seen that variables of age and marital status had significant adjusted associations with functional support (P = 0. 003 and P = 0.004, respectively) and structural support (P = 0.002 and P = 0.006, respectively). So that with increasing age, the scores of functional and structural support significantly increased (with adjusted for other variables in the model). The married people with physical disability compared to singles had higher levels of functional and structural supports. In the present study adjusted R2 values were 22% for functional support and 22% for structural support. 

**Table 3.** Multiple Linear Regression Model of Social Support Variables

Item	B	SE	P value
**Functional Support (Constant)**	1.63	.819	0.552
**Age**	1.069	.022	0.003
**Marital status**			
Single			
Married	3.36	.413	0.004
**Severity of disability**			
Mild			
Moderate	0.605	.544	0.358
Severe	0.58	.541	0.316
Very severe	1.41	.617	0.577

**Table 4.** Multiple Linear Regression Model of Socio-Demographic Variables

	B	SE	P value
**Structural support (Constant)**	0.829	1.463	0.824
**Gender**			
Female			
Male	1.61	.298	0.112
**Age**	1.06	.019	0.002
**Marital status**			
Single			
Married	2.74	.359	0.006
**Severity of disability**			
Mild			
Moderate	0.63	.472	0.344
Severe	0.60	.470	0.292
Very severe	1.28	.536	0.639

## 5. Discussion

Based on our literature review this study represents one of the first attempts to determine perceived social support status and its related factors in the physically disabled people in Iran. The results of this study showed the mean of different types of social support that derived from Norbeck questionnaire was respectively emotional support 90.24 (SD = 64.54), instrumental support 45.33 (33.1), functional support 135.57 (SD = 98.77) and structural support 77.37 (52.37). Given the attainable range of scores of various types of social support, it is not indicative of high levels of social support among participants.

The significant associations between marital status and functional and structural support (in favor of married participants) can be indicative of the extended social support network among married people with physical disability. They may have close relationship with spouse or spouse’s family and receive their support and attention. Thus, it appears that even though the majority of the participants in the study were single, and only a few were married, most of them had better supportive status, and hence had better understanding of the support from their network members. This finding suggested that not only physical disability for people is not restricted factor for marriage but also relationship with spouse as an important factor can influence on social support, well-being and quality of life. This finding is consistent with the result of other studies (22-25).

The regression model of social support showed that contrary to expectations there was no significant association between severity of disability and types of social support. It must be mention, in terms of this result there was not any consistency in the literature. However this result was in agreement with the results of previous studies (26). The results of another study did not consistent with it (27). This inconsistency may be due to the differences in sampling and tools that used in some studies. For example, unlike the present study that was conducted on the physically disabled people of both sexes (male and female) and regardless to type of disability, study of Friend et al. (2002) was conducted only on women with disabilities of rheumatoid arthritis. Also, their findings showed only significant correlation between variable of Social companionship and severity of disability (27) Also, in a study by Brien et al., study population consisted of patients with multiple sclerosis, which was different from the participants in the present study (18) and this may be a reason for disagreement of the two studies. The variable of socioeconomic status had no significant correlation with any of the dimensions studied. This could be interpreted in that the socioeconomic status of the disabled in the present study did not influence their perceived social support at any level. However, it must be mention that, given the socioeconomic status measuring method in this study, this variable may don’t truly reflect the people’s socioeconomic status (28), and this shows the need for further study for accurate assessment of the relationship between these two variables.

Evidence reveals that employment status is associated with social support of the physically disabled. However, in the present study, no significant correlation was found between this variable and any of the variables of social support, neither in bivariate analysis nor in the regression model. In addition to intrinsic differences associated with samples, these anomalies could reflect problems in different methodologies (different sample size, different tools, and different study designs). Also, the above finding about employment status may be due to the unemployed status of the majority of the participants (66.9%) compared to the employed participants (33.1%). Also, with considering lacking of correlation between having insurance and different types of social support, it must be mentioned that the majority of the participants (78.7%) had insurance, and only a few had not (21.3%). Thus, due to low variance in insurance variable, no significant difference showed among participants in terms of insurance variable. In the present study, significant correlation was observed between age variable and functional and structural supports, which agrees with the results of other studies (16, 27), and the physically disabled people reported higher perceived social support with increasing age.

### 5.1. Limitations

First, this was a cross-sectional study, thus, the observed association cannot be interpreted as causal inferences. Second, because this study targeted physically disabled people, who were covered by welfare organization with the mentioned condition, the findings cannot be generalized to other physically disabled people. Third, in this study, perceived social support was investigated, and with tools used for social support, the quality and quantity of social support cannot be distinguished.

### 5.2. Strong Points

The Positive points in this study were: estimate of perceived social support status among people with physical disability and adjusted associations between perceived social support and related factors with using multiple regression models.

### 5.3. Conclusion

Given the level of various social supports found in this study, participants didn’t have proper social support status (in any dimension). Given that social support as a social determinant of health has an important role in improving psychosocial adjustment and well-being in people’s lives, knowledge of social support in different group of society, especially in this vulnerable group, is important. Also, the variables that remained in linear regression model (age, marital status) can indicate that in interpreting social support and using suitable interventions of physically disabled people, cultural and social factors in society must be considered. Another limitation is self-report measures.
